# Innovation and Technology in Iran

**Published:** 2017

**Authors:** Shahin Akhondzadeh

Scientific production of Iran’s scientists holds the needed potential and wealth to be the world’s reference in science and knowledge. Based on this and by following guidelines of the supreme leader of the Islamic revolution, preservation, persistence, and reinforcement of the scientific production debate has unavoidable urgency and priority in academic and scientific circles and gatherings. We must support innovative and technological institutes more than before.

Even though the country has achieved regional first rank in number of published articles and enjoys noticeable ranking in the world either in basic or in clinical medicine ^1,2^, it seems the process of commercializing articles and other research products and transforming research into product and innovation production is still in need of more attention. It is such that Iran’s rank in international innovation indicators, or Global Innovation Index, is still not noticeable. Despite holding first rank in the region for article publication and scientific production, in regards to global innovation indicators, Iran holds 11^th^ rank in the region, ranking which places Iran after countries like United Arab Emirates and Kuwait. In this ranking system, unfortunately, Iran holds rank of 78 among the 143 countries of the world. In the same ranking system, Iran’s rank in the world in regards to infrastructure for innovation is 91, in regards to creative output is 75, and in regards to knowledge and technology output is 65. It seems, in this field, more drive and effort should be put forth to shape a system for transforming science into innovation, commercializing research and pivoting research on production so Iran can be transformed into an innovative country with an economy pivoted on science and also in the innovation arena can acquire needed authority.
